# Bougie-in-channel intubation technique

**DOI:** 10.1186/s13054-018-2184-9

**Published:** 2018-10-06

**Authors:** Kay Choong See, Melanie Estaras, Rolando Capistrano, Sui Hua Wong, Juliet Sahagun, Juvel Taculod

**Affiliations:** 1 Division of Respiratory and Critical Care Medicine, University Medicine Cluster, National University Hospital, 1E Kent Ridge Road, NUHS Tower Block Level 10, Singapore, 119228 Singapore; 2 Division of Critical Care, National University Hospital, 1E Kent Ridge Road, NUHS Tower Block Level 10, Singapore, 119228 Singapore

Dear Editor,

Compared with elective endotracheal intubation for surgery, emergency endotracheal intubation for critically ill patients is more difficult because of the poor cardiorespiratory reserve of patients, stress and urgency of intubation, and environmental limitations outside of the operation theatre setting. Therefore, emergency intubation has higher complication rates and lower first-attempt success [1, 2]. Methods used to facilitate intubation include videolaryngoscopes and bougies [2, 3], and bougie use improves first-attempt intubation success compared with stylet use [4].

Although videolaryngoscopy often improves vocal cord visualization, intubation success does not seem to improve when adding videolaryngoscopy to bougie use. Better visualization does not ensure easier intubation, as insertion of the bougie into the trachea would still require optimal neck positioning and a straight oral-pharyngeal-laryngeal axis [5]. Channeled blades attached to hyperangulated videolaryngoscopes can overcome the limitation of neck positioning as the channel can guide a bougie toward the glottic opening. The bougie then can be shifted laterally out of the channel, allowing a large (size 7 or greater) endotracheal tube to be railroaded and rotated easily.

**Fig. 1:**
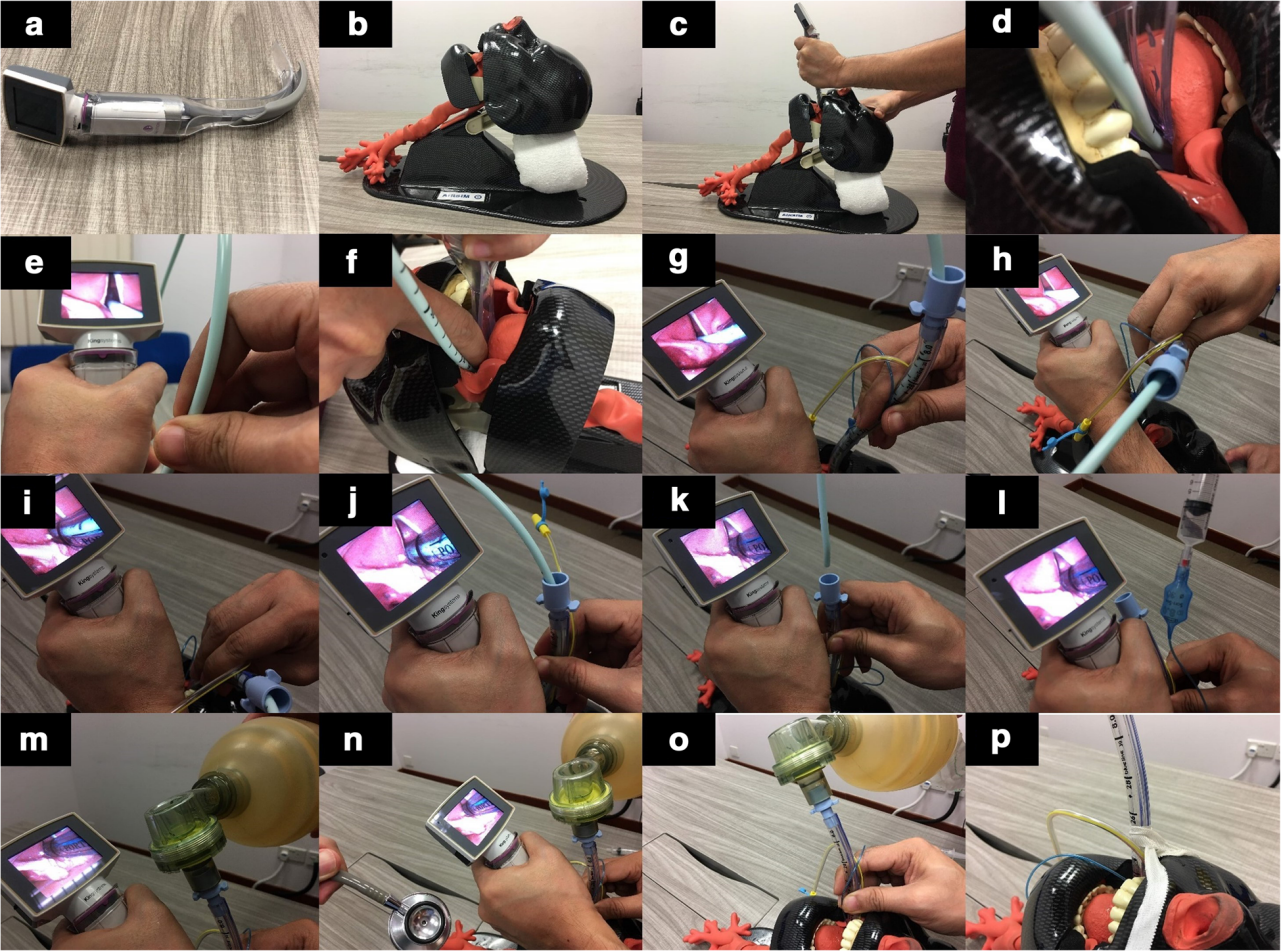
Bougie-in-channel intubation technique. **a** Attach the disposable channeled blade to the videolaryngoscope. **b** Allow the patient’s neck to be in the neutral position. **c** Insert the videolaryngoscope into the oral cavity and visualize the glottic opening. **d** Insert the flexible bougie into the guiding channel of the disposable videolaryngoscope blade (that is, bougie-in-channel technique). **e** Advance the flexible bougie into the trachea. **f** While holding the videolaryngoscope in place, shift the bougie laterally out of the channel. **g** While holding the videolaryngoscope in place, railroad a lubricated endotracheal tube down the bougie until resistance is felt at the arytenoids and then pull the endotracheal tube back by about 2 cm. **h** While holding the videolaryngoscope in place, rotate the endotracheal tube 90° toward the patient’s left side (the bougie must be allowed to freely rotate together with the tube). **i** While holding the videolaryngoscope in place, advance the endotracheal tube until the black indicator line is at the level of the vocal cords. **j** While holding the videolaryngoscope in place, rotate the endotracheal tube back to the neutral position. **k** While holding the videolaryngoscope in place, remove the bougie. **l** While holding the videolaryngoscope in place, inflate the cuff. **m** While holding the videolaryngoscope in place, connect the tube to a ventilation device. **n** While holding the videolaryngoscope in place, confirm ventilation by chest auscultation. **o** Remove the videolaryngoscope. **p** Secure the endotracheal tube

Since June 2018, we started to use the channeled blade of the King Vision videolaryngoscope (Ambu A/S, Ballerup, Denmark) in the following 16-step fashion (Fig. 1). Twenty-five respiratory therapists were introduced to the bougie-in-channel technique via a 15-min tutorial and demonstration. Of these therapists, who have never performed endotracheal intubation before, 24 successfully intubated a manikin within 2 min each and one therapist intubated at a second attempt. This technique has been rolled out in our medical intensive care unit with first-attempt success among the first 10 patients, and a formal audit is in progress.
